# Malignant transformation of abdominal wall endometriosis with lymph node metastasis^☆^: Case report and review of literature

**DOI:** 10.1016/j.gynor.2013.12.003

**Published:** 2014-01-05

**Authors:** Francesc Fargas Fàbregas, Maite Cusidó Guimferrer, Francesc Tresserra Casas, Sonia Baulies Caballero, Rafael Fábregas Xauradó

**Affiliations:** Instituto Universitari Dexeus, Barcelona, Spain

**Keywords:** Malignant endometriosis, Lymph node metastasis

## Abstract

• A simple endometriosis can result in malignancy pathology, as a neoplasia.
• Wall-abdominal tumors and soft tissue as a possible differential diagnosis of abdominal wall endometriosis
• Preperitoneal node-metastasis as malignancy of endometriosis in previous cesarean scar

## Introduction

Endometriosis is defined as the emergence and growth of endometrial tissue (glands and stroma) outside of the uterus. It affects 8–15% of the population and is most frequently found in the ovary. Extrapelvic implantation may occur in any organ or tissue, but is rare in the abdominal wall. When it does occur there, a history of surgery, whether laparotomy or laparoscopy, is usually present.

Malignancy of this type of endometriosis in an abdominal wall scar may occur from just a few months until up to 18 years after surgery (Hensen et al., 2006). The case we discuss below deals with the malignant transformation of cesarean scar endometriosis with nodal metastasis at preperitoneal level. This is interesting because no other known cases have ever been reported.

## Case presentation

We herein present the case of a 49 year-old patient with a history of appendectomy, two births, a cesarean section, and removal of the endometriotic mass in the cesarean scar, 15 years before.

The patient was seen for her routine annual gynecologic examination, in which she reported the presence of a painful abdominal mass, which had increased in size during the previous few months. She described the pain as cyclical, corresponding to her menstrual cycles, and that it reminded her of the lesion for which she had previously been operated on. Her check-ups prior to this consultation had been normal and no abdominal nodule had been observed.

A physical examination revealed a mass located in the midline of the abdominal wall, 3 cm above the Pfannenstiel scar. The size of the lesion was roughly 6 cm × 6 cm, and it was painful upon movement and was Fothergill sign positive. The rest of the gynecological examination was normal.

**Fig. 1:**
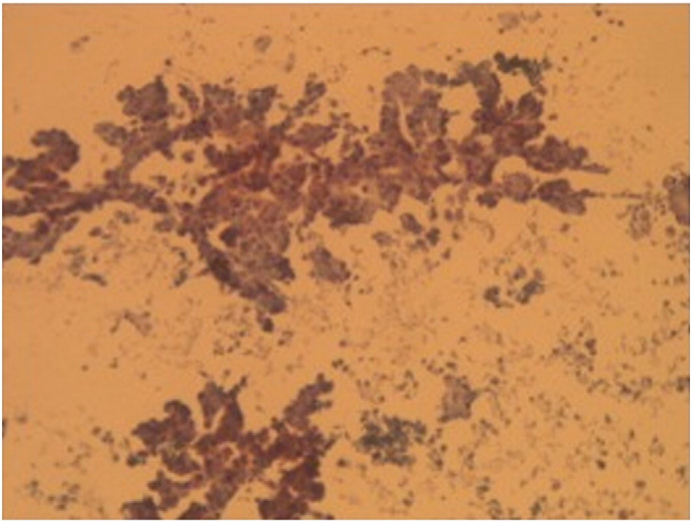
Citological study.

Ultrasound scan and mammography were normal. Soft tissue ultrasound (abdominal wall) revealed a lesion measuring 70 × 60 mm located in the abdominal wall, confined within the mesogastrium, caudal to the umbilical region, compatible with fibrous soft tissue tumor (desmoid tumor). Due to the history of the patient we were not able to rule out endometriotic implants. Fine-needle aspiration was performed with pap smear revealing a benign process compatible with endometriosis (Fig. 1).

Tumor marker levels were: CA-125: 243 U/mL and CEA: < 0.5 ng/mL. With a diagnosis of abdominal wall endometriosis, surgery was scheduled for removal of the wall tumor and exploration of the abdominal cavity.

## Surgical procedure

**Fig. 2:**
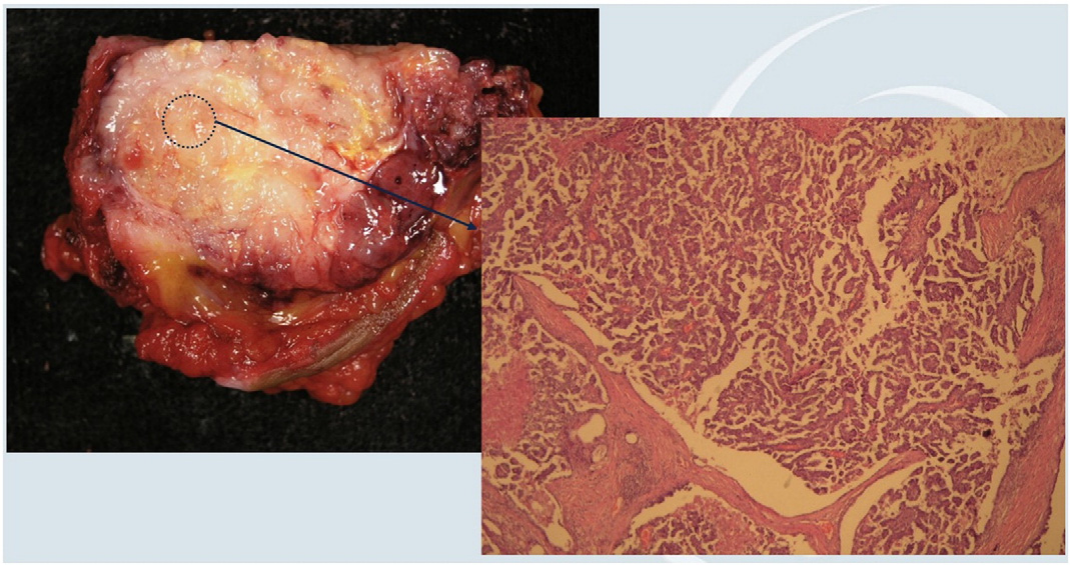
Pathological study of the tumor (macroscopic and microscopica study).

The intraoperative pathology of the abdominal wall nodule revealed a mass located at the preperitoneal level with histopathologic result of serous papillary adenocarcinoma (Fig. 2). During the exploratory laparotomy two preperitoneal nodules were observed in the right iliac fossa and another two in the left iliac fossa, which were resected and observed to be nodal metastases of serous papillary adenocarcinoma (two right and one left). The surgery proceeded with bilateral adnexectomy, omentectomy, a removal of the endometriotic mass located in the uterine fundus, endometrial biopsy, and a thorough assessment of the abdominal cavity with intraoperative examination of all the specimens, none revealing neoplastic infiltration.

The case was evaluated by the Gynecology and Oncology Committee, which decided that an extension study with a PET/CT was to be performed. Two hypermetabolic images were observed, one at the level of the abdominal wall mass and another one in the splenic flexure. Surgical laparoscopy was performed with removal of the splenic flexure, which was reported as steatonecrosis with chronic inflammation. The fibrotic area in the abdominal wall mass was also resected, with the pathology providing a benign result as well.

Subsequently, adjuvant treatment was administered with 6 cycles of carboplatin and Taxol, plus quarterly follow-ups for clinical examination and laboratory tests (CA-125), and semi-annual follow-ups for radiological tests. After a 48-month follow-up the patient is disease-free.

## Discussion

The probability of developing endometriosis in a surgical scar is roughly 0.03–1% (Hensen et al., 2006; Blanco et al., 2003) and malignant transformation is likely to occur in 0.3 to 1% of the cases (Matter et al., 2003; Leng et al., 2006), 80% of which are located in the ovary. The principal risk factors of malignant transformation of endometriosis include: advanced age of the patient, if they are postmenopausal, and if the tumor diameter of an endometriotic lesion is > 9 cm (Kobayashi et al., 2007). The probability that it exclusively affects the rectus abdominis muscles without peritoneal infiltration or aponeurosis is also rather low, there have only been 18 well-described cases, and there haven't been any cases published at all documenting extraperitoneal nodal metastases (Gianella et al., 2010).

Abdominal scar endometriosis presents as a mass in the abdominal wall, usually adjacent to a scar from previous surgery, and is painful, with or without increased size of the scar. The abdominal pain described by the patient is usually cyclical, correlating with her menstrual cycles, if hormonal reserve is still present (Hensen et al., 2006).

Surgical history must be taken into account for the proper diagnosis. Imaging techniques can also be useful. As a first choice, soft tissue ultrasound is recommended, complementing it with ultrasound-guided fine-needle aspiration of the mass. When there is a suspected diagnosis, as in our case (due to the history of the patient), serum levels of CA-125 are requested. Non-invasive diagnostic tests have a very low sensitivity in such cases.

In spite of all these tests, the definitive histopathological diagnosis was serous papillary adenocarcinoma.

The differential diagnosis with palpable masses in the abdominal wall should include: hernia, hematoma, lymphadenopathy, lymphoma, lipoma, abscess, subcutaneous cyst, neuroma, soft tissue sarcoma, desmoid tumor or metastasis.

The most frequent extragonadal sites of malignant endometriosis are (> 50% of the cases): the rectovaginal septum, the colon and the vaginal wall (Bats et al., 2008). In our case, the tumor was located in the preperitoneal space, affecting the rectus abdominis muscles. Therefore, the Fothergill sign was not useful in differentiating it from a rectus sheath hematoma or any other pathology exclusively affecting the abdominal wall muscles.

The most interesting aspect of the case is the nodal metastases, since it is the first case described in literature. Being a preperitoneal tumor, the nodal metastases follow the pattern of tumor spread that affects the abdominal wall muscles. When the primary lesion is located in the infraumbilical region, these metastases drain to the superficial inguinal chains. When the lesion is in the supraumbilical region, nodal metastatic spread may drain to the axillary chains.

CA-125 levels in the blood might be slightly elevated however it is a less specific biomarker. Many studies have evaluated different biomarkers for predicting or excluding endometriosis.

Recently, one interesting study showed that CRP serum levels were significantly higher in patients affected by endometriosis than in healthy patients. Similarly, other studies have shown that patients with mild endometriosis had lower anti-Müllerian hormone levels compared to patients without disease.

Another interesting and innovative study demonstrated an increase in follistatin serum levels in patients with endometrioma when compared to patients affected by other types of benign ovarian cysts.

Studies are also underway regarding other probable and more specific biomarkers of ovarian carcinoma and endometriosis, such as the HE4 protein (human epididymis protein 4) combined with CA-125.

Evidently, surgery is the definitive therapeutic approach. In our case, the patient was scheduled for surgery under the assumption of a benign diagnosis (abdominal wall endometrioma) in order to resect the mass and thoroughly examine the abdominal cavity.

The majority of carcinomas derived from endometriosis are of endometrioid or clear cell histologic type, in some series accounting for 70% of the subtypes (Modesitt et al., 2002). Our case was a serous papillary carcinoma, malignized from an endometriotic lesion in a cesarean scar. Although intracavitary malignization is well known, extraperitoneal malignization is not so common. In addition to the peculiarity of this finding, the absence of a primary ovarian lesion and the presence of extraperitoneal nodal metastasis make this a one-of-a-kind case.

Several molecular factors have been found to be involved in the association of endometriosis with ovarian cancer. They include: p53 alterations, PTEN silencing, K-Ras mutations and even expression of HNF-1β (especially in clear cell tumors). It's known that the micro-environments of endometriosis and endometriosis associated ovarian cancer share similar cytokines and mediators. Whether this similarity represents a link between endometriosis and endometriosis associated ovarian cancer or simply an employment of common signaling molecules to two separate lesions remains to be seen (Worley et al., 2013). The molecular mechanisms, however, remain unclear.

**Fig. 3:**
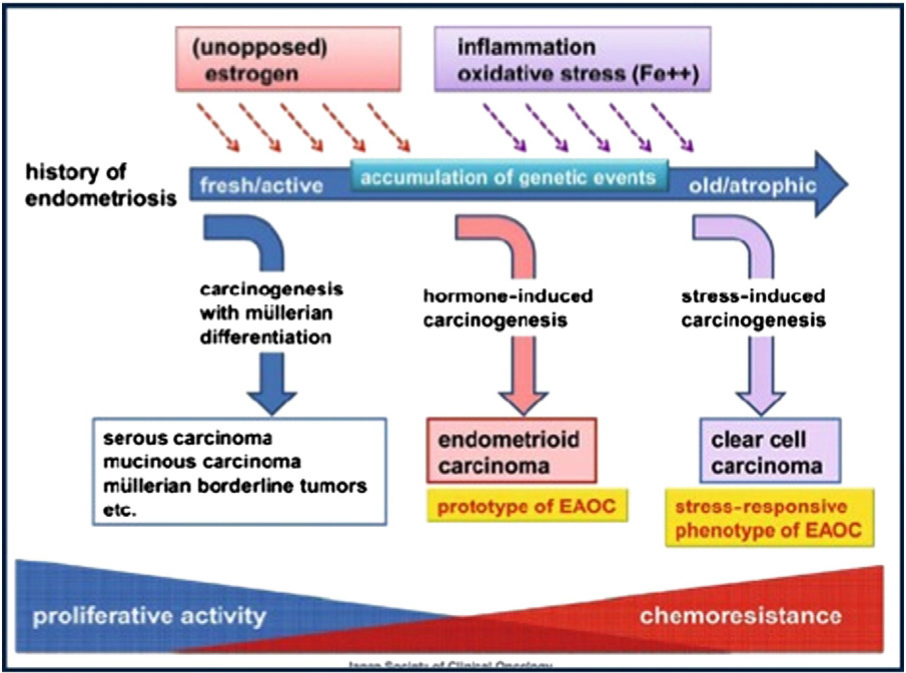
Endometriosis carcinogenesis.

One hypothetical model describing the pathogenesis of malignant transformation of endometriosis is from the Japanese Society of Clinical Oncology, which examines three simple and typical mechanisms of carcinogenesis (Fig. 3).

The first speculates that the endometriotic epithelium serves as a precursor of cancer in the same way that the surface epithelium of a normal ovary gives rise to ovarian tumors and contributes to the emergence of epithelial tumors, epithelial–stromal tumors, both, or borderline tumors.

The second mechanism refers to similarities in the carcinogenic process of endometrial neoplasia, which, under the influence of unopposed estrogens, basically leads to an endometrioid tumor.

The third describes the idea that this carcinogenesis is strongly influenced by the microenvironment within the endometriotic lesion and that continued exposure to oxidative stress and inflammation promotes a resistant and slow-growing phenotype of malignant tumors such as clear cell carcinoma.

Although this hypothesis may be simplistic and require further validation, it remains a useful model of preliminary carcinogenic mechanisms in endometriosis (Mandai et al., 2009).

These patients cannot be treated in the same way as those with ovarian neoplasia, since this is not the lesion to be treated, nor does it have the same dissemination mechanism. The literature primarily describes surgical treatment, the surgical removal of the disease, while ruling out metastasis in possible organs or epithelia at risk. For this reason we decided to remove all the macroscopically visible disease along with the omentum, both ovaries and an endometrial biopsy.

As for the adjuvant therapy, the patient was treated with carboplatin and Taxol because serous papillary histologies tend to respond to this combination of chemotherapy.

## Conclusions

This is a unique case since no other well-documented cases of malignant transformation of endometriosis with preperitoneal lymph node metastasis have been described in literature. Because this is such a rare etiology, there is very little experience in the management of these types of patients to help guide us in considering an ideal treatment strategy. Further molecular studies are needed to shed light on the mechanisms of the pathogenesis of this disease. Nevertheless, we are certain that the proper treatment of endometriosis, or the monitoring of these patients, can either prevent the very occurrence of a malignant lesion or allow for diagnosis and treatment at an early stage.

## Conflict of interest statement

The authors declare that there is no conflict of interest.
